# 1-Butyl-3-(1-naphthyl­meth­yl)benzimidazolium hemi{di-μ-iodido-bis­[diiodidomercurate(II)]} dimethyl sulfoxide monosolvate

**DOI:** 10.1107/S1600536809046376

**Published:** 2009-11-21

**Authors:** Zhi-Qiang Wang, Gang Shen, Zhan-Ying Zheng, Xiu-Mei Wu, Qing-Xiang Liu

**Affiliations:** a Tianjin Key Laboratory of Structure and Performance of Functional Molecules, College of Chemistry and Life Science, Tianjin Normal University, Tianjin 300387, People’s Republic of China; b State Key Laboratory of Element-Organic Chemistry, Nankai University, Tianjin 300071, People’s Republic of China

## Abstract

In the title compound, (C_22_H_23_N_2_)[Hg_2_I_6_]_0.5_·(CH_3_)_2_SO, the 1-butyl-3-(1-naphthyl­meth­yl)benzimidazolium anion lies across a centre of inversion. The dihedral angle between the benzimidazolium and naphthalene ring systems is 81.9 (3)°. In the crystal structure, π–π stacking inter­actions are observed between the imidazolium ring and the unsubstituted benzene ring of the naphthalene ring system, with a centroid–centroid separation of 3.510 (5) Å. In the centrosymmetric anion, the Hg(II) atoms are in a distorted tetrahedral coordination. The dimethyl sulfoxide solvent mol­ecule is disordered over two sites with occupancies of 0.615 (9) and 0.385 (9).

## Related literature

For background to the chemistry of imidazolium compounds, see: Arduengo *et al.* (1991 ▶); Garrison & Youngs (2005 ▶). For a related structure, see: Liu *et al.* (2003 ▶).
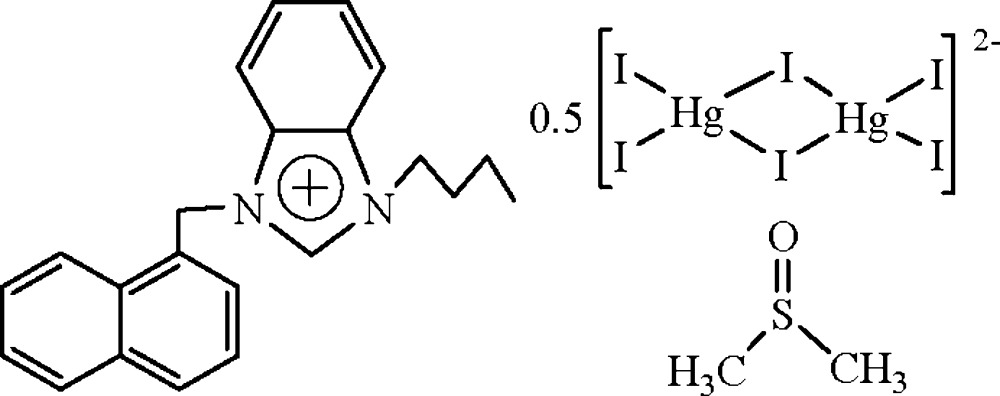


## Experimental

### 

#### Crystal data

(C_22_H_23_N_2_)[Hg_2_I_6_]_0.5_·C_2_H_6_OS
*M* *_r_* = 974.85
Monoclinic, 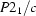

*a* = 12.0565 (13) Å
*b* = 13.7312 (16) Å
*c* = 18.378 (2) Å
β = 102.089 (2)°
*V* = 2975.0 (6) Å^3^
*Z* = 4
Mo *K*α radiation
μ = 8.38 mm^−1^
*T* = 296 K
0.28 × 0.26 × 0.22 mm

#### Data collection

Bruker SMART CCD area-detector diffractometer
Absorption correction: multi-scan (*SADABS*; Sheldrick, 1996 ▶) *T*_min_ = 0.110, *T*_max_ = 0.158
14876 measured reflections
5253 independent reflections
3641 reflections with *I* > 2σ(*I*)
*R*_int_ = 0.044

#### Refinement

*R*[*F* ^2^ > 2σ(*F* ^2^)] = 0.043
*wR*(*F* ^2^) = 0.111
*S* = 1.03
5253 reflections
327 parameters
60 restraints
H-atom parameters constrained
Δρ_max_ = 1.16 e Å^−3^
Δρ_min_ = −1.71 e Å^−3^

### 

Data collection: *SMART* (Bruker, 1998 ▶); cell refinement: *SAINT* (Bruker, 1998 ▶); data reduction: *SAINT*; program(s) used to solve structure: *SHELXS97* (Sheldrick, 2008 ▶); program(s) used to refine structure: *SHELXL97* (Sheldrick, 2008 ▶); molecular graphics: *SHELXTL* (Sheldrick, 2008 ▶); software used to prepare material for publication: *SHELXTL*.

## Supplementary Material

Crystal structure: contains datablocks global, I. DOI: 10.1107/S1600536809046376/ci2946sup1.cif

Structure factors: contains datablocks I. DOI: 10.1107/S1600536809046376/ci2946Isup2.hkl

Additional supplementary materials:  crystallographic information; 3D view; checkCIF report

## Figures and Tables

**Fig. 1.**
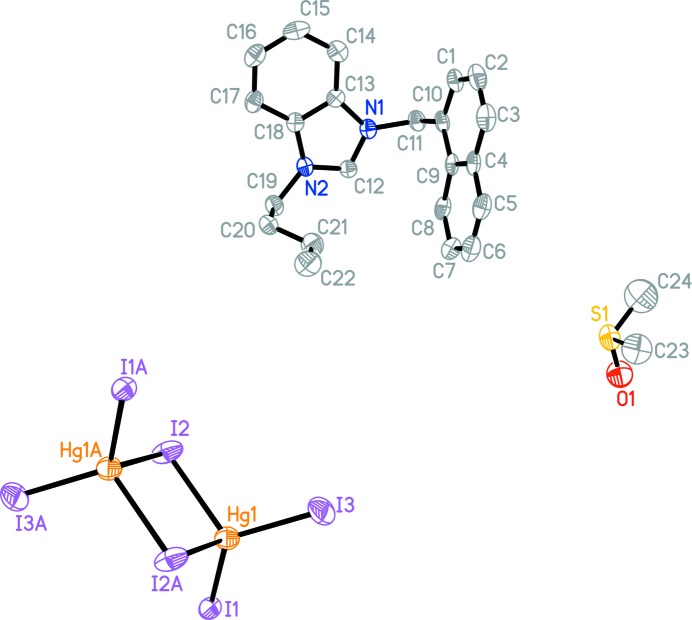
The asymmetric unit of the title compound, with displacement ellipsoids drawn at the 30% probability level. H atoms have been omitted for clarity. Only the major component of the disordered DMSO molecule is shown.

## supplementary crystallographic information

### Comment

Since the discovery of free stable N-heterocyclic carbene (NHC) (Arduengo *et al.*, 1991), the carbene chemistry based on imidazol-2-ylidene (imy) or benzimidazol-2-ylidene (bimy) have been receiving considerable attention, owing to their inherent stability, their interesting characteristics of structure and bonding, and their potential for synthesis and as catalysts in organic reactions (Garrison & Youngs, 2005). We report here the synthesis and crystal structure of the title compound (Fig. 1).

The dihedral angle between benzimidazolium and naphthalene ring systems is 81.9 (3)°. The butyl group and 1-naphthylmethyl group lie on the same side of benzimidazole ring system. The N—C and C—N—C angles agree with those observed in 1-(9-anthracenylmethyl)-3-ethylimidazolium iodide (Liu *et al.*, 2003).

The [Hg_2_I_6_]^2-^ anionic unit lies across a centre of inversion, in which atoms Hg1, I2, Hg1A and I2A are coplanar, with I2—Hg1—I2A and Hg1—I2—Hg1A angles of 94.29 (2)° and 85.71 (2)°, respectively. All Hg—I bond distances fall in the regular range of 2.6792 (9)–2.8943 (4) Å.

### Experimental

A solution of 1-iodobutane (1.583 g, 8.6 mmol) and 1-(1-naphthylmethyl)benzimidazole (1.860 g, 7.2 mmol) in THF (100 ml) was stirred for five days under refluxing, and a pale yellow precipitate was formed. The product was filtred and washed with THF. The powder of benzimidazolium iodide are obtained by recrystallization from methanol/diethyl ether [3.5 g (91%), m.p 188–190°C]. A mixture of benzimidazolium iodide (0.199 g, 0.45 mmol) and mercury(II) iodide (0.245 g, 0.54 mmol) in THF (20 ml) and dichloromethane (20 ml) was refluxed for 24 h. A brown solution was formed and the solvent was removed with a rotary evaporator. Water (30 ml) was added to the residue and the solution was extracted with CH_2_Cl_2_ (30 ml). The extracting solution was dried with anhydrate MgSO_4_, then the solution was concentrated to 10 ml and diethyl ether (5 ml) was added. A pale yellow powder was obtained, and isolation by filtration yielded the title compound [yield 0.401 g (83%), m.p 235–237°C). ^1^H NMR (300 MHZ, DMSO-d6): 0.97 (t, J = 5.4, 3H, CH_3_), 1.46 (m, 2H, CH_2_), 2.03 (m, 2H, CH_2_), 4.55 (t, J = 7.2, 2H, CH_2_), 6.31 (s, 2H, CH_2_), 7.43 (t, J = 6.0, 1H, PhH), 7.55 (d, J = 6.0, 2H, PhH), 7.63 (d, J = 6.3, 4H, PhH), 7.65 (t, J = 8.8, 1H, PhH), 7.91 (d, J = 8.4, 2H, PhH), 8.13 (d, J = 8.4, 1H, PhH), 11.23 (s, 1H, bimiH) (bimi: benzimidazole). Single crystals suitable for X-ray diffraction were obtained by slow evaporation of an CH_2_Cl_2_ solution at room temperature.

### Refinement

The dimethyl sulfoxide solvent molecule is disordered over two sites with occupancies of 0.615 (9) and 0.385 (9). U^ij^ and distance restraints were applied. All H atoms were initially located in a difference Fourier map. They were then placed in geometrically idealized positions and constrained to ride on their parent atoms, with C*sp*^3^–H = 0.97 Å, C*sp*^2^–H = 0.93 Å and *U*_iso_(H) = 1.2*U*_eq_(C). The highest residual density peak is located 0.95 Å from atom I1 and the deepest hole is located 0.79 Å from atom I2.

### Crystal data

**Table:**

(C_22_H_23_N_2_)[Hg_2_I_6_]_0.5_·C_2_H_6_OS	*F*(000) = 1800
*M**_r_* = 974.85	*D*_x_ = 2.177 Mg m^−^^3^
Monoclinic, *P*2_1_/*c*	Mo *K*α radiation, λ = 0.71073 Å
Hall symbol: -P 2ybc	Cell parameters from 3658 reflections
*a* = 12.0565 (13) Å	θ = 2.3–23.9°
*b* = 13.7312 (16) Å	µ = 8.38 mm^−^^1^
*c* = 18.378 (2) Å	*T* = 296 K
β = 102.089 (2)°	Block, yellow
*V* = 2975.0 (6) Å^3^	0.28 × 0.26 × 0.22 mm
*Z* = 4	

### Data collection

**Table:**

Bruker SMART CCD area-detector diffractometer	5253 independent reflections
Radiation source: fine-focus sealed tube	3641 reflections with *I* > 2σ(*I*)
graphite	*R*_int_ = 0.044
φ and ω scans	θ_max_ = 25.0°, θ_min_ = 1.7°
Absorption correction: multi-scan (*SADABS*; Sheldrick, 1996)	*h* = −14→14
*T*_min_ = 0.110, *T*_max_ = 0.158	*k* = −16→11
14876 measured reflections	*l* = −21→21

### Refinement

**Table:**

Refinement on *F*^2^	Primary atom site location: structure-invariant direct methods
Least-squares matrix: full	Secondary atom site location: difference Fourier map
*R*[*F*^2^ > 2σ(*F*^2^)] = 0.043	Hydrogen site location: inferred from neighbouring sites
*wR*(*F*^2^) = 0.111	H-atom parameters constrained
*S* = 1.03	*w* = 1/[σ^2^(*F*_o_^2^) + (0.0474*P*)^2^ + 6.0193*P*] where *P* = (*F*_o_^2^ + 2*F*_c_^2^)/3
5253 reflections	(Δ/σ)_max_ = 0.001
327 parameters	Δρ_max_ = 1.16 e Å^−^^3^
60 restraints	Δρ_min_ = −1.71 e Å^−^^3^

### Special details

**Table:**

Geometry. All e.s.d.'s (except the e.s.d. in the dihedral angle between two l.s. planes) are estimated using the full covariance matrix. The cell e.s.d.'s are taken into account individually in the estimation of e.s.d.'s in distances, angles and torsion angles; correlations between e.s.d.'s in cell parameters are only used when they are defined by crystal symmetry. An approximate (isotropic) treatment of cell e.s.d.'s is used for estimating e.s.d.'s involving l.s. planes.
Refinement. Refinement of *F*^2^ against ALL reflections. The weighted *R*-factor *wR* and goodness of fit *S* are based on *F*^2^, conventional *R*-factors *R* are based on *F*, with *F* set to zero for negative *F*^2^. The threshold expression of *F*^2^ > σ(*F*^2^) is used only for calculating *R*-factors(gt) *etc*. and is not relevant to the choice of reflections for refinement. *R*-factors based on *F*^2^ are statistically about twice as large as those based on *F*, and *R*- factors based on ALL data will be even larger.

### Fractional atomic coordinates and isotropic or equivalent isotropic displacement parameters (Å^2^)

**Table:**

	*x*	*y*	*z*	*U*_iso_*/*U*_eq_	Occ. (<1)
Hg1	0.61164 (3)	0.57544 (3)	0.95810 (2)	0.07147 (16)	
I1	0.83044 (5)	0.51958 (4)	0.98219 (4)	0.06220 (19)	
I2	0.47338 (6)	0.40737 (6)	0.90484 (4)	0.0883 (3)	
I3	0.53530 (6)	0.73713 (6)	0.88235 (5)	0.0985 (3)	
N1	−0.0121 (6)	0.4144 (4)	0.2680 (3)	0.0446 (15)	
N2	0.0684 (5)	0.3180 (4)	0.3578 (3)	0.0433 (15)	
C1	−0.1740 (8)	0.6136 (6)	0.2289 (5)	0.059 (2)	
H1	−0.2223	0.5783	0.1922	0.071*	
C2	−0.2176 (9)	0.6929 (7)	0.2618 (6)	0.071 (3)	
H2	−0.2938	0.7092	0.2468	0.085*	
C3	−0.1503 (9)	0.7452 (7)	0.3148 (5)	0.065 (3)	
H3	−0.1804	0.7969	0.3370	0.078*	
C4	−0.0332 (8)	0.7225 (6)	0.3373 (4)	0.051 (2)	
C5	0.0393 (9)	0.7759 (6)	0.3924 (5)	0.063 (2)	
H5	0.0099	0.8269	0.4158	0.076*	
C6	0.1503 (10)	0.7553 (7)	0.4125 (5)	0.071 (3)	
H6	0.1966	0.7916	0.4495	0.085*	
C7	0.1962 (9)	0.6798 (7)	0.3781 (5)	0.071 (3)	
H7	0.2732	0.6658	0.3922	0.085*	
C8	0.1289 (7)	0.6259 (6)	0.3235 (5)	0.057 (2)	
H8	0.1614	0.5769	0.2999	0.068*	
C9	0.0119 (7)	0.6431 (5)	0.3025 (4)	0.0476 (19)	
C10	−0.0635 (7)	0.5867 (5)	0.2488 (4)	0.0460 (19)	
C11	−0.0249 (7)	0.4955 (6)	0.2146 (4)	0.052 (2)	
H11A	−0.0801	0.4782	0.1700	0.063*	
H11B	0.0470	0.5078	0.2005	0.063*	
C12	0.0855 (7)	0.3871 (5)	0.3116 (4)	0.0431 (18)	
H12	0.1560	0.4131	0.3096	0.052*	
C13	−0.0978 (6)	0.3588 (5)	0.2866 (4)	0.0412 (17)	
C14	−0.2127 (8)	0.3544 (6)	0.2578 (5)	0.058 (2)	
H14	−0.2478	0.3943	0.2189	0.069*	
C15	−0.2726 (8)	0.2872 (8)	0.2901 (6)	0.069 (3)	
H15	−0.3506	0.2823	0.2728	0.083*	
C16	−0.2195 (9)	0.2262 (7)	0.3481 (6)	0.070 (3)	
H16	−0.2632	0.1813	0.3677	0.084*	
C17	−0.1077 (8)	0.2305 (6)	0.3763 (5)	0.057 (2)	
H17	−0.0729	0.1902	0.4151	0.068*	
C18	−0.0465 (7)	0.2981 (5)	0.3446 (4)	0.0445 (18)	
C19	0.1555 (8)	0.2795 (6)	0.4189 (4)	0.058 (2)	
H19A	0.2299	0.2893	0.4078	0.070*	
H19B	0.1442	0.2101	0.4239	0.070*	
C20	0.1505 (8)	0.3299 (6)	0.4918 (5)	0.059 (2)	
H20A	0.0752	0.3207	0.5016	0.071*	
H20B	0.2039	0.2981	0.5315	0.071*	
C21	0.1762 (9)	0.4371 (7)	0.4943 (5)	0.072 (3)	
H21A	0.2527	0.4460	0.4867	0.086*	
H21B	0.1250	0.4684	0.4532	0.086*	
C22	0.1665 (9)	0.4881 (8)	0.5648 (5)	0.084 (3)	
H22A	0.0898	0.4837	0.5715	0.126*	
H22B	0.1870	0.5553	0.5619	0.126*	
H22C	0.2165	0.4579	0.6062	0.126*	
S1	0.5566 (4)	0.8983 (4)	0.1687 (3)	0.0831 (19)	0.615 (9)
O1	0.6734 (8)	0.9226 (16)	0.1578 (11)	0.132 (10)	0.615 (9)
C23	0.5144 (19)	0.9966 (15)	0.2185 (12)	0.127 (11)	0.615 (9)
H23A	0.5567	0.9953	0.2689	0.191*	0.615 (9)
H23B	0.4349	0.9912	0.2181	0.191*	0.615 (9)
H23C	0.5285	1.0568	0.1954	0.191*	0.615 (9)
C24	0.4652 (16)	0.921 (2)	0.0812 (8)	0.160 (13)	0.615 (9)
H24A	0.4795	0.8736	0.0457	0.241*	0.615 (9)
H24B	0.4790	0.9850	0.0645	0.241*	0.615 (9)
H24C	0.3877	0.9157	0.0861	0.241*	0.615 (9)
S1'	0.5614 (7)	0.9773 (6)	0.1314 (4)	0.089 (3)	0.385 (9)
O1'	0.6753 (11)	0.943 (2)	0.1700 (14)	0.100 (11)	0.385 (9)
C23'	0.481 (2)	0.995 (2)	0.2005 (13)	0.077 (9)	0.385 (9)
H23D	0.5145	1.0455	0.2339	0.116*	0.385 (9)
H23E	0.4789	0.9355	0.2278	0.116*	0.385 (9)
H23F	0.4046	1.0133	0.1772	0.116*	0.385 (9)
C24'	0.485 (2)	0.8777 (19)	0.0855 (15)	0.112 (12)	0.385 (9)
H24D	0.5197	0.8569	0.0454	0.168*	0.385 (9)
H24E	0.4083	0.8968	0.0659	0.168*	0.385 (9)
H24F	0.4866	0.8250	0.1200	0.168*	0.385 (9)

### Atomic displacement parameters (Å^2^)

**Table:**

	*U*^11^	*U*^22^	*U*^33^	*U*^12^	*U*^13^	*U*^23^
Hg1	0.0457 (2)	0.0806 (3)	0.0874 (3)	0.00266 (18)	0.01229 (19)	0.0094 (2)
I1	0.0479 (3)	0.0505 (3)	0.0878 (4)	0.0062 (3)	0.0134 (3)	−0.0007 (3)
I2	0.0773 (5)	0.1162 (6)	0.0770 (5)	−0.0384 (4)	0.0289 (4)	−0.0373 (4)
I3	0.0758 (5)	0.1001 (6)	0.1121 (6)	0.0222 (4)	0.0029 (4)	0.0270 (5)
N1	0.060 (4)	0.042 (4)	0.034 (3)	0.004 (3)	0.015 (3)	−0.001 (3)
N2	0.051 (4)	0.041 (3)	0.041 (4)	0.007 (3)	0.014 (3)	0.001 (3)
C1	0.075 (6)	0.050 (5)	0.053 (5)	0.005 (4)	0.013 (5)	0.016 (4)
C2	0.085 (7)	0.057 (6)	0.076 (7)	0.021 (5)	0.027 (6)	0.018 (5)
C3	0.099 (8)	0.047 (5)	0.060 (6)	0.015 (5)	0.039 (6)	0.002 (5)
C4	0.075 (6)	0.039 (4)	0.046 (5)	0.001 (4)	0.029 (4)	0.012 (4)
C5	0.091 (8)	0.048 (5)	0.059 (6)	−0.007 (5)	0.035 (5)	0.002 (4)
C6	0.108 (9)	0.053 (6)	0.057 (6)	−0.018 (6)	0.025 (6)	−0.002 (5)
C7	0.073 (6)	0.057 (6)	0.080 (7)	−0.015 (5)	0.012 (5)	0.009 (5)
C8	0.068 (6)	0.043 (5)	0.068 (6)	−0.002 (4)	0.036 (5)	0.010 (4)
C9	0.065 (5)	0.038 (4)	0.046 (5)	0.002 (4)	0.026 (4)	0.016 (4)
C10	0.070 (6)	0.043 (4)	0.028 (4)	0.008 (4)	0.014 (4)	0.009 (3)
C11	0.068 (5)	0.052 (5)	0.038 (4)	0.005 (4)	0.012 (4)	0.004 (4)
C12	0.047 (5)	0.044 (4)	0.038 (4)	0.000 (3)	0.009 (4)	−0.003 (4)
C13	0.046 (4)	0.042 (4)	0.039 (4)	0.003 (3)	0.016 (4)	−0.008 (4)
C14	0.069 (6)	0.055 (5)	0.050 (5)	0.009 (5)	0.014 (4)	−0.009 (4)
C15	0.047 (5)	0.086 (7)	0.075 (7)	−0.012 (5)	0.015 (5)	−0.027 (6)
C16	0.076 (7)	0.069 (6)	0.071 (6)	−0.017 (5)	0.029 (6)	−0.004 (5)
C17	0.071 (6)	0.055 (5)	0.049 (5)	−0.008 (4)	0.024 (5)	0.002 (4)
C18	0.057 (5)	0.042 (4)	0.038 (4)	0.003 (4)	0.017 (4)	−0.006 (4)
C19	0.064 (5)	0.053 (5)	0.056 (5)	0.006 (4)	0.008 (4)	0.007 (4)
C20	0.062 (5)	0.063 (6)	0.048 (5)	−0.004 (4)	0.002 (4)	0.010 (4)
C21	0.079 (7)	0.080 (7)	0.058 (6)	−0.003 (5)	0.018 (5)	0.004 (5)
C22	0.099 (8)	0.098 (8)	0.058 (6)	−0.015 (7)	0.022 (6)	−0.016 (6)
S1	0.078 (3)	0.085 (3)	0.094 (3)	0.016 (2)	0.036 (2)	0.019 (2)
O1	0.119 (11)	0.140 (11)	0.139 (11)	0.006 (5)	0.033 (5)	−0.008 (5)
C23	0.128 (12)	0.129 (12)	0.127 (12)	0.003 (5)	0.030 (5)	−0.004 (5)
C24	0.159 (13)	0.161 (13)	0.161 (13)	0.004 (5)	0.033 (6)	−0.001 (5)
S1'	0.093 (4)	0.086 (5)	0.098 (5)	0.007 (3)	0.043 (3)	0.009 (3)
O1'	0.090 (12)	0.107 (12)	0.108 (12)	0.004 (5)	0.028 (5)	−0.008 (5)
C23'	0.077 (10)	0.079 (10)	0.077 (10)	0.002 (5)	0.019 (5)	−0.004 (5)
C24'	0.110 (13)	0.111 (13)	0.114 (13)	0.002 (5)	0.024 (6)	−0.003 (5)

### Geometric parameters (Å, °)

**Table:**

Hg1—I3	2.6792 (9)	C15—C16	1.402 (13)
Hg1—I1	2.6933 (7)	C15—H15	0.93
Hg1—I2	2.8943 (5)	C16—C17	1.341 (12)
Hg1—I2	2.8943 (4)	C16—H16	0.93
I2—Hg1	2.8943 (4)	C17—C18	1.387 (11)
N1—C12	1.331 (9)	C17—H17	0.93
N1—C13	1.384 (9)	C19—C20	1.519 (12)
N1—C11	1.472 (9)	C19—H19A	0.97
N2—C12	1.318 (9)	C19—H19B	0.97
N2—C18	1.383 (9)	C20—C21	1.503 (12)
N2—C19	1.466 (10)	C20—H20A	0.97
C1—C10	1.357 (11)	C20—H20B	0.97
C1—C2	1.400 (13)	C21—C22	1.498 (13)
C1—H1	0.93	C21—H21A	0.97
C2—C3	1.339 (13)	C21—H21B	0.97
C2—H2	0.93	C22—H22A	0.96
C3—C4	1.420 (13)	C22—H22B	0.96
C3—H3	0.93	C22—H22C	0.96
C4—C5	1.400 (12)	S1—O1	1.5010 (9)
C4—C9	1.428 (11)	S1—C23	1.7650 (9)
C5—C6	1.342 (14)	S1—C24	1.7750 (9)
C5—H5	0.93	C23—H23A	0.96
C6—C7	1.387 (14)	C23—H23B	0.96
C6—H6	0.93	C23—H23C	0.96
C7—C8	1.368 (12)	C24—H24A	0.96
C7—H7	0.93	C24—H24B	0.96
C8—C9	1.403 (11)	C24—H24C	0.96
C8—H8	0.93	S1'—O1'	1.484 (10)
C9—C10	1.423 (11)	S1'—C24'	1.761 (10)
C10—C11	1.518 (11)	S1'—C23'	1.771 (9)
C11—H11A	0.97	C23'—H23D	0.96
C11—H11B	0.97	C23'—H23E	0.96
C12—H12	0.9300	C23'—H23F	0.96
C13—C14	1.376 (11)	C24'—H24D	0.96
C13—C18	1.392 (10)	C24'—H24E	0.96
C14—C15	1.380 (13)	C24'—H24F	0.96
C14—H14	0.93		
			
I3—Hg1—I1	122.65 (3)	C17—C16—H16	119.0
I3—Hg1—I2	111.97 (3)	C15—C16—H16	119.0
I1—Hg1—I2	107.66 (3)	C16—C17—C18	116.6 (9)
I3—Hg1—I2^i^	103.69 (3)	C16—C17—H17	121.7
I1—Hg1—I2^i^	112.94 (3)	C18—C17—H17	121.7
I2—Hg1—I2^i^	94.29 (2)	N2—C18—C17	131.3 (8)
Hg1—I2—Hg1^i^	85.71 (2)	N2—C18—C13	106.5 (6)
C12—N1—C13	108.0 (6)	C17—C18—C13	122.1 (8)
C12—N1—C11	124.8 (7)	N2—C19—C20	110.9 (7)
C13—N1—C11	127.1 (7)	N2—C19—H19A	109.5
C12—N2—C18	108.4 (6)	C20—C19—H19A	109.5
C12—N2—C19	124.8 (7)	N2—C19—H19B	109.5
C18—N2—C19	126.4 (7)	C20—C19—H19B	109.5
C10—C1—C2	122.1 (9)	H19A—C19—H19B	108.0
C10—C1—H1	118.9	C21—C20—C19	115.3 (8)
C2—C1—H1	118.9	C21—C20—H20A	108.4
C3—C2—C1	120.4 (9)	C19—C20—H20A	108.4
C3—C2—H2	119.8	C21—C20—H20B	108.4
C1—C2—H2	119.8	C19—C20—H20B	108.4
C2—C3—C4	120.6 (8)	H20A—C20—H20B	107.5
C2—C3—H3	119.7	C22—C21—C20	115.6 (9)
C4—C3—H3	119.7	C22—C21—H21A	108.4
C5—C4—C3	121.9 (8)	C20—C21—H21A	108.4
C5—C4—C9	119.0 (8)	C22—C21—H21B	108.4
C3—C4—C9	119.1 (8)	C20—C21—H21B	108.4
C6—C5—C4	121.6 (9)	H21A—C21—H21B	107.4
C6—C5—H5	119.2	C21—C22—H22A	109.5
C4—C5—H5	119.2	C21—C22—H22B	109.5
C5—C6—C7	120.1 (10)	H22A—C22—H22B	109.5
C5—C6—H6	119.9	C21—C22—H22C	109.5
C7—C6—H6	119.9	H22A—C22—H22C	109.5
C8—C7—C6	120.5 (10)	H22B—C22—H22C	109.5
C8—C7—H7	119.8	O1—S1—C23	106.2 (8)
C6—C7—H7	119.8	O1—S1—C24	105.2 (7)
C7—C8—C9	121.2 (9)	C23—S1—C24	98.3 (6)
C7—C8—H8	119.4	S1—C23—H23A	109.5
C9—C8—H8	119.4	S1—C23—H23B	109.5
C8—C9—C10	124.0 (7)	H23A—C23—H23B	109.5
C8—C9—C4	117.5 (8)	S1—C23—H23C	109.5
C10—C9—C4	118.6 (8)	H23A—C23—H23C	109.5
C1—C10—C9	119.2 (7)	H23B—C23—H23C	109.5
C1—C10—C11	118.8 (8)	S1—C24—H24A	109.5
C9—C10—C11	122.0 (7)	S1—C24—H24B	109.5
N1—C11—C10	110.3 (6)	H24A—C24—H24B	109.5
N1—C11—H11A	109.6	S1—C24—H24C	109.5
C10—C11—H11A	109.6	H24A—C24—H24C	109.5
N1—C11—H11B	109.6	H24B—C24—H24C	109.5
C10—C11—H11B	109.6	O1'—S1'—C24'	108.6 (8)
H11A—C11—H11B	108.1	O1'—S1'—C23'	107.1 (8)
N2—C12—N1	110.6 (7)	C24'—S1'—C23'	98.1 (7)
N2—C12—H12	124.7	S1'—C23'—H23D	109.5
N1—C12—H12	124.7	S1'—C23'—H23E	109.5
C14—C13—N1	132.3 (8)	H23D—C23'—H23E	109.5
C14—C13—C18	121.2 (8)	S1'—C23'—H23F	109.5
N1—C13—C18	106.5 (6)	H23D—C23'—H23F	109.5
C13—C14—C15	116.1 (8)	H23E—C23'—H23F	109.5
C13—C14—H14	122.0	S1'—C24'—H24D	109.5
C15—C14—H14	122.0	S1'—C24'—H24E	109.5
C14—C15—C16	122.0 (8)	H24D—C24'—H24E	109.5
C14—C15—H15	119.0	S1'—C24'—H24F	109.5
C16—C15—H15	119.0	H24D—C24'—H24F	109.5
C17—C16—C15	121.9 (9)	H24E—C24'—H24F	109.5
			
I3—Hg1—I2—Hg1^i^	−106.63 (3)	C18—N2—C12—N1	0.0 (8)
I1—Hg1—I2—Hg1^i^	115.73 (3)	C19—N2—C12—N1	−172.5 (6)
I2^i^—Hg1—I2—Hg1^i^	0.0	C13—N1—C12—N2	−0.7 (8)
C10—C1—C2—C3	0.0 (13)	C11—N1—C12—N2	174.5 (6)
C1—C2—C3—C4	1.3 (13)	C12—N1—C13—C14	−177.3 (8)
C2—C3—C4—C5	180.0 (8)	C11—N1—C13—C14	7.7 (12)
C2—C3—C4—C9	0.2 (12)	C12—N1—C13—C18	1.1 (8)
C3—C4—C5—C6	−178.7 (8)	C11—N1—C13—C18	−174.0 (6)
C9—C4—C5—C6	1.1 (12)	N1—C13—C14—C15	178.6 (7)
C4—C5—C6—C7	0.4 (13)	C18—C13—C14—C15	0.4 (11)
C5—C6—C7—C8	0.1 (14)	C13—C14—C15—C16	−0.8 (13)
C6—C7—C8—C9	−2.0 (13)	C14—C15—C16—C17	0.9 (14)
C7—C8—C9—C10	−176.8 (7)	C15—C16—C17—C18	−0.6 (13)
C7—C8—C9—C4	3.4 (11)	C12—N2—C18—C17	177.9 (8)
C5—C4—C9—C8	−2.9 (10)	C19—N2—C18—C17	−9.7 (13)
C3—C4—C9—C8	176.9 (7)	C12—N2—C18—C13	0.7 (8)
C5—C4—C9—C10	177.3 (7)	C19—N2—C18—C13	173.1 (7)
C3—C4—C9—C10	−2.9 (10)	C16—C17—C18—N2	−176.7 (8)
C2—C1—C10—C9	−2.8 (12)	C16—C17—C18—C13	0.1 (12)
C2—C1—C10—C11	175.5 (7)	C14—C13—C18—N2	177.5 (7)
C8—C9—C10—C1	−175.6 (7)	N1—C13—C18—N2	−1.1 (7)
C4—C9—C10—C1	4.2 (10)	C14—C13—C18—C17	−0.1 (11)
C8—C9—C10—C11	6.1 (11)	N1—C13—C18—C17	−178.7 (7)
C4—C9—C10—C11	−174.1 (6)	C12—N2—C19—C20	95.8 (9)
C12—N1—C11—C10	−97.1 (9)	C18—N2—C19—C20	−75.3 (9)
C13—N1—C11—C10	77.2 (9)	N2—C19—C20—C21	−63.9 (10)
C1—C10—C11—N1	−102.6 (8)	C19—C20—C21—C22	177.5 (8)
C9—C10—C11—N1	75.7 (9)		

Symmetry codes: (i) −*x*+1, −*y*+1, −*z*+2.
